# The Use of Potassium Chloride and Tapioca Starch to Enhance the Flavour and Texture of Phosphate- and Sodium-Reduced Low Fat Breakfast Sausages Manufactured Using High Pressure-Treated Meat

**DOI:** 10.3390/foods11010017

**Published:** 2021-12-22

**Authors:** Malco C. Cruz-Romero, Claire C. O’Flynn, Declan Troy, Anne M. Mullen, Joe P. Kerry

**Affiliations:** 1 Food Packaging Group, School of Food and Nutritional Sciences, University College Cork, T12 K8AF Cork, Ireland; 2 Teagasc, Food Research Centre, Ashtown, D15 KN3K Dublin, Ireland; O_claire@yahoo.com (C.C.O.); Declan.Troy@teagasc.ie (D.T.); anne.mullen@teagasc.ie (A.M.M.)

**Keywords:** high pressure processing, breakfast sausages, salt reduction, potassium chloride, tapioca starch, sensory evaluation

## Abstract

The objective of this study was to investigate the use of potassium chloride (KCl) and tapioca starch (TS) to reduce salt levels below 1.5% in sausages manufactured using previously high pressure (HP) processed pork (150 MPa). A 3 × 2 × 1 factorial design was used to formulate breakfast sausages with three salt levels (0.5%, 1.0%, and 1.5%), two ingredient levels (no added ingredient or added as a combination of KCl\TS), and one pressure level (150 MPa). Partial replacement of NaCl with KCl and addition of TS had beneficial effects on the water binding abilities of sausage batters by decreasing (*p* < 0.05) total expressible fluid (%) and increasing water holding capacity (%). Overall, results indicated that the use of KCl\TS imparted some beneficial effects to salt-reduced low fat breakfast sausages and has the potential to reduce salt levels in the breakfast sausages to 1.0% while still maintaining the organoleptic and functional properties traditionally associated with these meat products.

## 1. Introduction

Consumers are increasingly interested in anything that improves their quality of life and sodium reduction has been recognised by the meat industry as an important driver for consumers [1]. Diet may not be the only factor determining welfare and health, but it is certainly one of the most important; therefore, there is an increasing demand for safer and healthier food products [2]. Salt performs different functions in processed meat products, including enhancement of flavour, preservation through reduction in water activity (a_W_) values, masking of off-flavours, stabilization of product structure and texture, promotion of water-holding capacity, and enhancement of meat binding [3,4,5]. There are a number of health and safety concerns surrounding the manufacture of processed meat products, as processed meat products generally contain high salt levels; therefore, processed meat manufacturers require new solutions in the reformulation of low-sodium processed meat products as a means of reducing dietary sodium in consumer foods. This is a current industry target and a topic of intense international interest [6,7,8]. The World Health Organization (WHO) has recommended that food manufacturers reduce salt content in their food products, and in terms of product reformulation, the primary focus should be on the highest salt reduction possible [9]. The excessive consumption of sodium and high amounts of saturated fatty acid and cholesterol through diet has been related to hypertension and consequently to the increased risk of development of cardiovascular diseases [10,11]. Large health benefits to society may result from efforts to lower sodium consumption by modest amounts over time; however, a greater variety of low salt products must be offered to the consumer, especially in relation to processed meat products.

Meat batters are complex systems consisting of solubilised muscle proteins, muscle fibres, fragmented myofibrils, fat cells, fat droplets, water, salts, phosphates, and other ingredients and in meat formulations 1.5 to 2.0% salt is typically used to allow for the extraction and solubilisation of myofibrillar proteins [12]. In comminuted meat products, potassium chloride (KCl) is the most commonly used salt as a replacement for NaCl as it is a generally recognized as safe (GRAS) substance [13]. Monahan and Troy [14] reported that complete replacement of NaCl with KCl causes flavour problems; however, partial replacement (35–50%) can be accomplished without a loss of functionality and can enhance meat product flavour, while still fulfilling the low sodium requirements of these products [14,15]. Conversely, Schoene et al. [16] reported that a partial NaCl replacement generated sensory changes in odour, taste, and consistency of processed meat products; however, problems associated with the partial replacement of NaCl with KCl related more to sensory attributes than to textural attributes. Irrespective of the research conducted to date, masking the undesirable sensory attributes associated with the use of KCl remains a problem. The main issue associated with the use of KCl is the astringent flavour that accompanies its use in processed meat products.

A variety of non-meat ingredients have commonly been used as fillers, binders, and extenders in comminuted meat products to reduce cook-shrink and formulation costs, as well as to improve emulsifying capacity, emulsion stability, water binding potential, nutritive value, and slicing characteristics. Starch has traditionally been used in meat products to improve quality, the effect being based on the ability of starch to gelatinise when heated in a water-containing medium [17]. Tapioca starch, in the form of a modified tapioca starch product known as Tapiocaline™, is traditionally used in low-fat meat products to improve juiciness and succulence. However, it could also be beneficial when used in the formulation of low-salt products, where the solubility of the proteins is decreased due to low ionic strength. Shand et al. [18] reported that starches can be useful water-binding and texture modifying ingredients in low-salt systems, where the functionality of the myosin heat set matrix may be impaired due to the reduced ionic environment.

It has been shown that high-pressure (HP) processing of isolated bovine myofibrillar proteins induced the protein to adopt a new structure reminiscent of the molten globule state. Given that functional properties such as gelling, emulsifying, and foaming are highly linked with the structure of proteins, these properties are modified by HP processing [19]. HPP has been used to compensate for the reduction of sodium ion content in processed meats [20,21,22]. In our previous studies, HPP of meat at 150 MPa for 5 min at ambient temperature has shown great potential for reducing phosphate and salt levels (NaCl), which are meat processing ingredients required for adequate binding, without significantly affecting the organoleptic and functional properties traditionally associated with breakfast sausages [23,24]. While a general reduction in salt concentration to 1.5% was possible, it was noted that salt reduction in breakfast sausages below 1.5% had detrimental effects on both sensory and textural aspects. Therefore, the objective of this study was to investigate whether other functional ingredients such as a combination of KCl along with tapioca starch (TS) (KCl\TS), would aid in the reduction of salt levels below 1.5% in sausages manufactured using previously HP processed pork.

## 2. Materials and Methods

### 2.1. Breakfast Sausage Manufacture

Fresh pork belly (skinless and boneless) was obtained from a local meat processing plant. Excess fat was trimmed from the meat, cut into strips, and minced through a 5-mm plate using a Mainca mincer PM-82 (Maquinaria Industria Carnica, Barcelona, Spain). The minced pork was then mixed in a Mainca mixer RM.90 to obtain a homogeneous mass. Six batches of sausages were made in duplicate in the form of a 3 × 2 × 1 factorial design (Table 1). This incorporated three salt levels (0.5%, 1.0% and 1.5%); two ingredient levels (no addition, or addition of a combination of KCl\TS), and one pressure (150 MPa) (Table 1). The batches formulated to contain the ingredient KCl\TS had one third of the total NaCl concentration added as KCl (Merk extra pure food grade, Lennox suppliers, Dublin, Ireland). Heat treated Tapioca starch Tapiocaline EX533 (Fibrisol Sevices Ltd., London, UK), with its coarse texture (0.58 mm), was added at 3.0% of the total batch weight. Meat used to manufacture the breakfast sausages was HP-treated at 150 MPa for 5 min in an isostatic press (Engineered Pressure Systems International N.V, Temse, Belgium) as described previously by [24] and phosphate levels were kept constant at 0.25% [23]. The breakfast sausage batter was made as previously described by O’Flynn et al. [24] in a bowl chopper Mainca CR.22 (Equipamientos Cárnicos S.L. (Mainca), Barcelona, Spain). The batter was stuffed into 28 mm diameter collagen casings (Devro, McDonnells, Dublin, Ireland) using a Mainca piston filler. The sausages were hand linked and frozen until required for analysis.

### 2.2. Cooking Procedure

Sensory, texture, cooked compositional analysis, and instrumental colour measurement were performed on cooked samples as previously outlined by O’Flynn et al. [24]. Briefly, an electric grill was preheated at 200 °C for 5 min and then sausages cooked, turning at 5 min intervals, until an internal temperature of 77 °C was reached. Final internal end-point temperature was re-checked using a hand-held food thermometer.

### 2.3. Compositional Analysis and Emulsion Stability

Moisture, fat, and protein were determined on both the raw sausage batter and the cooked sausages. Fat and moisture were determined by an automated, integrated microwave moisture and dichloromethane fat extraction method using a CEM Meat Analysis System (CEM Corporation, Matthews, NC, USA) and protein by LECO organic nitrogen determinator (Model FP-528, LECO Corporation, St. Joseph, MI, USA) while emulsion stability, ash, and salt content were measured on raw batter as previously described by O’Flynn et al. [23]. The salt contents of raw batter were determined from the ashed samples by titration using silver nitrate (AgNO_3_). The percentage of salt present in raw sausage batter samples was calculated as follows:

$$\;\mathrm{Salt}\;\left(\%\right)=\frac{\mathrm{Titre}\;\mathrm{for}\;\mathrm{sample}\;\left(\mathrm{mL}\right)-\mathrm{Titre}\;\mathrm{for}\;\mathrm{blank}\;\left(\mathrm{mL}\right)}{\mathrm{Sample}\;\mathrm{weight}\;\left(g\right)}\times {\;\mathrm{Molarity}\;\mathrm{of}\;\mathrm{AgNO}}_{3}\times 5.844\;$$

The volume of total expressible fluid (TEF) and the percentage fat were calculated as follows:

$$\begin{array}{c} \mathrm{TEF}=\left(\mathrm{Weight}\;\mathrm{of}\;\mathrm{centrifuge}\;\mathrm{tube}\;\mathrm{and}\;\mathrm{sample}\right)-\left(\mathrm{Weight}\;\mathrm{of}\;\mathrm{centrifuge}\;\mathrm{tube}\;\mathrm{and}\;\mathrm{pellet}\right)\\ \mathrm{TEF}\;\left(\%\right)=\frac{\mathrm{TEF}}{\mathrm{Sample}\;\mathrm{Weight}}\times 100\\ \mathrm{Fat}\;\left(\%\right)=\frac{\left(\mathrm{Weight}\;\mathrm{of}\;\mathrm{Crucible}\;+\;\mathrm{Dried}\;\mathrm{Supernantant}\right)\;-\;\left(\mathrm{Weight}\;\mathrm{of}\;\mathrm{empty}\;\mathrm{Crucible}\right)}{\mathrm{TEF}}\times 100\; \end{array}$$

### 2.4. Water Holding Capacity

Five sausages per treatment were thawed overnight at 4 °C and WHC of each sample determined as outlined by O’Flynn et al. [24]. Briefly, 10 g of sausage was weighed into a glass jar, covered with lids and then heated for 10 min at 90 °C in a waterbath. After heating, samples were removed from the jar and cooled down for 30 min at room temperature. After cooling, each sample was wrapped in cheesecloth, placed into a centrifuge tube with cotton wool at the bottom, and centrifuged for 10 min at 10,000 rpm. The samples were removed from the centrifuge and re-weighed. The WHC (%) was calculated as follows:

$$\mathrm{WHC}\;\left(\%\right)=1-\frac{B-A}{M_{1}}\times 100=1-\frac{B-A}{M_{2}\times \;B}$$

B = Weight of sample before heating. A = Weight of sample after heating and centrifuging. M_1_ = Total water content in the meat sample. M_2_ = % moisture of the sample.

### 2.5. Cook Loss

The cook loss (%) of the sausages was determined by weight difference before and after cooking of 5 sausages per batch as previously described by O’Flynn et al. [24]. Each cooked sausage was patted dry with a paper towel to remove excess water before weighing.

### 2.6. Texture Profile Analysis

Cylindrical sections of the cooked breakfast sausages (diameter = 2.8 cm; height = 2 cm) were analysed at room temperature using an Instron Universal Testing Machine (model 4464 Instron Engineering Corp., Canton, MA, USA) as described by O’Flynn et al. [23]. The samples were subjected to compression using a 500 N load cell. The samples (5 cylindrical sections) were compressed to 50% of its original height with a 35 mm diameter cylindrical probe at a cross-head speed of 50 mm/min. Texture profile parameters measured were hardness (N), springiness (mm), adhesiveness (N), cohesiveness (dimensionless), gumminess (N), and chewiness (N-mm).

### 2.7. Colour

The colour of the cross-section of the cooked breakfast sausages was measured using a CIE Lab system with a dual beam xenon flash spectrophotometer (UltraScan XE, Hunterlab, Reston, VA, USA) as described by O’Flynn et al. [23].

### 2.8. Sensory Evaluation

An eight internally-trained taste panel was used to evaluate the breakfast sausages using a six point hedonic and intensity scale as previously outlined by O’Flynn et al. [24]. The panellists were chosen from a pool of 16 trained assessors based on their experience of sensory analysis of meat products and on their availability. Each cooked breakfast sausage was labelled with a 3-digit random number and four random samples per session were served to each of the 8 panellists. The tested attributes included: saltiness, juiciness, overall flavour intensity, overall firmness, other flavours, overall texture and overall acceptability. “Other” flavours were described to the panellists as non-meat and non-fat. Sensory evaluation was performed on all six treatments on eight separate occasions using eight independent sensory panels.

### 2.9. Statistical Analysis

The software STATGRAPHICS^®^ centurion XV (Statgraphics Technologies, Inc., The Plains, VA, USA) was used for the statistical analysis. For the compositional analysis, results were tested using a one-way analysis of variance (ANOVA) and significance assessed using Tukey’s test at 5% significance level. For the remaining experiments, the results were compared using a two-way ANOVA with salt level and added ingredient (KCl\TS) as factors. The difference between means of pairs was resolved by means of confidence intervals using least significance difference (LSD), the level of significance was set at *p* < 0.05. The whole experiment was repeated twice.

## 3. Results and Discussion 

### 3.1. Gross Composition Analysis of HP-Treated Breakfast Sausages

The effects of the treatments on the composition of the raw and cooked sausages are presented in Table 2. Adding the functional ingredient KCl\TS at each salt level decreased (*p* < 0.05) the moisture content in the raw sausage. This may be due to the fact that the percentage water added in the formulation was reduced to compensate for addition of these ingredients. Additionally, a significant (*p* < 0.05) decrease in moisture was noticed when the salt level was increased from 0.5 to 1.5% without the addition of KCl\TS; however, in samples containing 1.0% salt levels the moisture content was not significantly different to the raw sausage batter containing 0.5% salt levels (Table 2). The differences in added water to product batches containing 0.5% and 1.0% salt may not have been appreciable enough to cause a significant difference in % moisture. 

Fat content in raw batters ranged from 9.12 to 9.81% and differences in fat levels between treatments were not significant. This is consistent with results reported by O’Flynn et al. [23,24] where changing salt levels did not affect fat content. Ash content increased (*p* < 0.05) when the salt content increased and with the addition of KCl\TS. In cooked breakfast sausages, moisture content decreased when the ingredient KCl\TS was employed; however, in batches containing 0.5% salt, this difference was not significant. The fat content of the cooked breakfast sausages was higher than determined for raw breakfast sausages, as would be expected. The cooked breakfast sausages containing 1.5% salt and added KCl\TS had a lower fat content compared to raw product fat content (Table 2). Mittal and Barbut [25] reported that during cooking some moisture and fat is expelled from the product due to melting of fat and evaporation of moisture. The cooked breakfast sausages containing 0.5% NaCl without added KCl\TS had a lower (*p* < 0.05) protein content than those samples containing the same NaCl level but with the added ingredient KCl\TS. However, this trend was not observed for the other salt levels employed. As observed in raw breakfast sausage samples, reducing salt levels from 1.0 to 0.5% in cooked breakfast sausages which contained KCl\TS increased protein content. The observed differences in protein content may have been due to the different amounts of water added to the formulations under investigation; a decrease in added water may cause a detectable increase in the percentage of measured fat and protein. Crehan et al. [20] found an increase in protein content when salt levels were reduced from 2.5 to 1.5% in cooked frankfurters treated with 150 MPa. In this study, an increase in protein content observed after cooking may be due to decreased water levels and a slight decrease in the total product mass during cooking.

Salt content results revealed that salt levels determined in sausages were a little higher than targeted values. Replacing one third of the NaCl with KCl and the addition of TS at the 1.0% salt level resulted in a decrease (*p* < 0.05) in sodium content compared to the sausages formulated to contain 1.0% NaCl and without the addition of KCl\TS (Table 2). However, this result is not consistent, as the same trend was not observed for product batches formulated to contain 0.5 or 1.5% NaCl. Ash content for the sample containing 0.5% NaCl increased with the use of KCl\TS and additionally, there was a significant (*p* < 0.05) increase in ash content due to each higher salt level used. Desmond [26] stated that in processed meats, in addition to NaCl, several ingredients used also contain sodium in substantial amounts and in a typical meat product containing 2% salt, NaCl contributes approximately 79% of the sodium present in the final meat product. The other ingredients that contain sodium are sodium nitrite, sodium nitrate, sodium phosphates, sodium ascorbate and erythorbate, monosodium glutamate, hydrolysed vegetable protein, non-fat dry milk, sodium caseinate, whey blends, soy proteins, water, and natural casings. This may explain the higher than expected values for the salt contents of the manufactured sausages as some of these ingredients were included in the final sausage formulations.

### 3.2. Emulsion Stability

The percentage of total expressible fluid (TEF) decreased (*p* < 0.05) with the addition of the KCl\TS; however, the emulsion stability was not significantly affected by adjustment of salt level (% TEF and % Fat) or by the addition of KCl\TS (Table 3). This is consistent with the results reported by Yang et al. [27] who showed that HP-treated reduced fat and salt sausages (200 MPa for 2 min at 10 °C) had better emulsion stability with reduced fat losses compared to untreated samples. Smith [12] stated that the main purpose of starch addition in a meat emulsion is to bind water. The significantly lower %TEF for the samples with added KCl\TS suggested that a more stable gel matrix was formed, thereby improving the stability of the batter. It has been reported that some extender materials have a detrimental effect on batter stability because they compete for available water, thereby making it unavailable for involvement in the protein-water matrix and resulting in its reduced effectiveness [28]. These authors also indicated that higher values for emulsion stability (due to binders) were not necessarily indicative of a more desirable appearance in the finished product. Gordon and Barbut [29] reported no difference in fat loss when 1.5% NaCl was substituted with KCl to provide equal ionic strengths.

### 3.3. Cook Loss

Cooking loss data are critical to the meat industry as a means of predicting the behaviour of the product during cooking as cook loss can directly affect the juiciness of the meat product after cooking [23]. Cook loss was significantly (*p* < 0.05) affected by treatments. The addition of KCl\TS did not contribute to a high yield if salt levels were reduced. Without the addition of these ingredients, salt levels could be reduced from 1.5 to 1.0% without causing significant increases in cook loss (Table 3). Results showed that when sausages were manufactured with 0.5% NaCl, then cook losses were significantly reduced (*p* < 0.05) with the addition of KCl\TS (Table 3). A decreased cook loss was reported when tapioca starch was added to meat products [30,31]. Sikes et al. [22] reported that pressure treatment at 200 MPa for 2 min at 10 °C significantly reduced cooking losses in batters through the use of just 0.5% added salt, whereas, it was determined that 1% salt was required to reduce cooking losses by about 75% when no pressure treatment was employed. Mor-Mur and Yuste [32] reported that HP-treated sausages (500 MPa for 5 or 15 min at 65 °C) had lower cook losses and higher yields compared to heat treated sausages (40 min at 80 or 85 °C).

It was reported that salt levels in frankfurters and breakfast sausages manufactured using HP treated meat could be reduced to 1.5% without significantly affecting cooking losses [20,24]. However, employing salt levels below 1.5% significantly increased the cook loss of breakfast sausages manufactured with meat HP treated at 150 MPa [24]. Conversely, it has been also reported that HP treatment of reduced fat and salt sausages and pork patties with addition of 1% NaCl had significantly lower water or cooking losses compared to untreated samples [27,33]. Ruusunen et al. [34] reported that modified tapioca starch decreased frying losses and improved water and fat binding in low-salt frankfurters. Kim et al. [35] reported that the functional properties of added ingredients in low\reduced-salt meat emulsion systems are the most important factors affecting water and binding properties.

### 3.4. WHC

Water holding capacity is the ability of meat to retain its water in the sample when external forces such as heating, pressing or grinding are applied [36]. Protein matrix stability is not only essential to the texture of the product, but essential to the water and fat holding capacities of the comminuted meat system. WHC was interactively affected by the treatments of salt level and KCl\TS addition (Table 3). The interactive effect showed that it was possible to reduce salt levels to 0.5% without negatively affecting the WHC of sausages, provided that KCl\TS was present in the formulation; however only a salt reduction to 1.0% was possible without the addition of these ingredients. Results showed that the addition of KCl\TS assisted in the solubilisation of salt soluble proteins in low salt sausages, thereby improving WHC. Starch has been described as an ingredient which can improve the water binding properties of meat batters [37] due to its high swelling capacity. The gel matrix formed with alternative binders differs from that when meat is mixed with sodium chloride, as they provide bind through a combination of protein coagulation and gel formation rather than direct interaction with the muscle proteins [38]. Nisar et al. [31] reported that cooking yield increased as TS increased in the formulation of low-fat buffalo meat patties. Chan et al. [39] reported that HP treatment (50–100 MPa) of meat used for the manufacture of processed meat products with reduced salt content improved water holding capacity. These authors proposed that owing to pressurisation effects, the resulting meat had higher protein surface hydrophobicity with greater exposure of sulfhydryl groups and increased protein solubility. Sun and Holley [40] reported that HP treatment at relatively low levels increases the solubility of myofibrillar protein and promotes depolymerization, thereby improving meat gelation. However, treatment temperature, salt concentration, and fat level can also influence HP processing effects on meat gelation properties.

### 3.5. Sensory Analysis

The effects of salt content and addition of KCl\TS on the average sensory scores for breakfast sausages are presented in Table 4. In general, the salt level used in sausage formulation had a significant (*p* < 0.05) effect on all sensory attributes examined, with the exception of other flavours. However, none of the sensory attributes examined were affected by the addition of KCl\TS and there was no interactive effect between salt level used and addition of KCl\TS. 

Salt concentration in breakfast sausages, not surprisingly, affected (*p* < 0.05) saltiness perception (Table 4). The scores provided by the taste panellists for saltiness were significantly (*p* < 0.05) reduced with each treatment reduction in NaCl concentration. Estevez et al. [41] also found that NaCl concentration directly influenced saltiness in seabass sausages. The fact that there were no sensory changes following the addition of KCl\TS suggests that when NaCl concentration was substituted with KCl, the concentration used may have been too low to have had an effect on the perceived saltiness of the sausages. This indicates that it would be possible to reduce the sodium content of sausages, without affecting saltiness, through the replacement of one third of the NaCl with KCl. Claus and Hunt [42] also found no effect on saltiness due to the addition of 3.5% starch in bologna sausage. Paulsen et al. [43] studied the effects of different salt substitutes (KCl, Na-lactate, K-lactate/Na-diacetate and milk minerals) on the sensory perception of sausages and reported that the samples with KCl substitution were most similar to the control sample; described by high intensity of fattiness, juiciness, flour flavour, and brief dominance of meat flavour. 

Juiciness was not significantly affected by a salt reduction to 1.0% (Table 4). It was expected that the addition of TS would increase the juiciness of sausages as the manufacturer product specification states that TS could bind 3- to 5-times its own weight in water, thereby retaining moisture during cooking. However, the results indicated that juiciness decreased but not significantly. Similarly, Claus and Hunt [41] reported a decrease in juiciness due to the use of starch. This decrease in juiciness may have been due to the fact that TS bonded strongly with water and subsequently, caused the sausage to appear dry to panellists. Tobin et al. [5] reported a positive correlation of juiciness to salt content from 2.4 to 1.6%; however, a significant negative correlation at lower salt levels of 1.4 to 0.8%. This may be due to increased levels of extracted and solubilised myofibrillar proteins at higher salt levels [5,12].

The flavour scores for cooked breakfast sausages decreased (*p* < 0.05) when salt levels were reduced below 1.5%; however, flavour scores were not significantly reduced due to the addition of KCl\TS (Table 4). Blandness in meat products owing to the use of starches has been widely reported [17,44]. Doyle and Glass [6] reported that NaCl affects the taste of foods by providing the flavour of saltiness, by enhancing or masking other flavours and by controlling growth of microbes that produce flavourful compounds. It is unlikely that KCl, at its low level of addition, would have imparted an unusual or bitter flavour (sometimes associated with the use of KCl) on the sausages. In general, it was reported that a substitution above a concentration of 50% of NaCl with KCl produces unacceptable sensory scores and masking the undesirable sensory attributes associated with potassium chloride remains a problem [15].

When salt levels were reduced below 1.0%, there was a decrease (*p* < 0.05) in the overall firmness of sausages (Table 4). Hand et al. [45] found a decrease in firmness when 35% of NaCl was replaced with KCl. In this study, a decrease in firmness was also observed following the addition of ingredients, but this decrease was not significant. In general, meat fillers have beneficial effects upon emulsion stability and yield, but negative effects on product firmness [46]. This lower firmness may be due to a less homogeneous and grainier structure. Other flavours, described to the panellists as non-meat and non-fat, were not significantly affected by any of the treatments. However, scores for other flavours were reduced when salt levels were increased or when KCl\TS was not included in the specific formulation (Table 4).

The overall texture of cooked breakfast sausages was not affected by the addition of the functional ingredient KCl\TS, however, overall texture scores for cooked breakfast sausages was reduced (*p* < 0.05) with each reduction in salt level from 1.5% (Table 4). Annor-Frempong et al. [44] found improved emulsion-type pork sausages consistency and texture following the use of cassava starch and this improvement in texture may be attributed to the optimal gel-forming and pasting properties of cassava [47]. Olson and Terrell [48] found no difference in the texture of bologna sausage made using NaCl or that made using a mixture of NaCl and KCl.

The overall acceptability scores for cooked breakfast sausages decreased significantly when salt levels were reduced below 1.5% (Table 4). Olson and Terrell [48] found a small, but non-significant, decrease in the acceptability of fresh pork sausage made using 0.45% NaCl and 0.45% KCl compared to a sausage made with 1.25% NaCl. The authors suggested that a gradual decrease in the NaCl content of meat products would allow consumers adapt to a blander taste, allowing KCl to be used as a partial functional replacement for NaCl. Mor-Mur and Yuste [32] reported that from a sensory perspective, the sensory panel did not detect differences between HP-treated or heat-treated sausages; and when there were differences, pressurised samples were preferred on more occasions because of their better appearance, taste, and especially texture.

### 3.6. Texture Profile Analysis

The texture profile of the cooked breakfast sausages is shown in Table 5. An interaction effect between salt level and the addition of KCl\TS was observed for hardness. Hardness, adhesiveness, cohesiveness, and chewiness were the textural attributes affected (*p* < 0.05) by the level of salt used; however, the factor addition of KCl\TS affected (*p* < 0.05) only the textural attributes adhesiveness and cohesiveness. Sikes et al. (2009) reported that the use of moderate pressures around 200 MPa for 2 min at 10 °C were most effective in producing cooked beef batters with reduced salt contents, together with high quality textural properties. In addition to the benefits of reduced cooking losses, various textural attributes, such as; hardness, fracture force, gumminess and chewiness were all improved.

Texture profile analysis results (Table 5) indicated that salt level can be reduced to 1.0% without affecting hardness or springiness when KCl\TS was added. In general, in order to produce an acceptable texture in cooked sausage products, 1.8 to 2% salt content is required in meat emulsions to achieve extraction of salt-soluble protein [22]. Cohesiveness decreased significantly (*p* < 0.05) with decreasing salt level and the presence of KCl\TS. Gumminess decreased (*p* < 005) when salt level decreased; however, no significant gumminess effect was observed when KCl\TS was added. The poor texture associated with low-salt meat products (1% salt) has been one of the primary reasons why low-salt meat products have failed to be commercialised successfully. Sikes et al. [22] reported that using HPP on meat batters containing 1% NaCl produced products with similar, if not better, texture and cook yield compared to non-HPP samples containing 2% salt. Iwasaki et al. [33] reported that HP-treatment at 200 MPa prior to heating increased the apparent elasticity of pork patties; however, pressure treatment above 200 MPa decreased the apparent elasticity. The apparent elasticity of pressure-treated (200 MPa) pork patties was doubly higher compared to untreated control samples [33] and sausages containing 1% NaCl HP-treated at 200 MPa before heating, showed the highest apparent elasticity. Mor-Mur and Yuste [32] reported that HP-treated sausages were more cohesive and less firm compared to heat treated sausages.

The results from this study (Table 5) show that the effect of KCl\TS addition was dependent upon the salt content of the batters. Salt levels can be reduced to 1.0% with or without the use of KCl\TS; however, sausages made with KCl\TS and 1.0% salt were harder (*p* < 0.05) than those made using 1.0% NaCl alone. Mandava et al. [49] found that the addition of 2% modified potato starch to low-fat sausages increased the firmness of non-pressure-treated sausages and samples HP-treated at 100 MPa. Pressure-treated samples, both with and without starch, had improved firmness values and cook yields. Fernandez et al. [50] examined the effects of many ingredients, including the addition starch, on the texture of pressurised and non-pressurised chicken meat batters. They reported that the addition of starch to chicken meat batters increased hardness values due to a decrease in the protein/moisture ratio, rather than by a contribution to a stronger heat-induced structure, and which was brought about by the swelling of starch granules in the protein gel matrix [51]; thus producing harder structures with better water binding capabilities. However, a comparison of batter’s pressure treated at 200 MPa showed that the addition of starch did not affect hardness values [50]. Differences between poultry and pork batters may account for finding variations reported between this study and that by Fernandez et al. [50]. Land et al. [52] stated that the functional properties of myosystems vary according to species and this may influence, not only the characteristics of the products, but also their responses to a specific process such as the application of high pressure [53].

Independent of the addition of KCl\TS, there was no significant difference in the springiness of the cooked sausages when salt levels were reduced from 1.5 to 0.5% (Table 5). Similar results were found by Fernandez et al. [50] following the addition of starch to pressure-treated chicken batters.

Cooked sausage adhesiveness was not significantly affected when salt levels were reduced from 1.5 to 1.0% (Table 5). However, the addition of KCl\TS increased (*p* < 0.05) the adhesiveness of the cooked sausages. Hung and Smith [54] reported that carbohydrates are often added as another gelling system in meat products to enhance texture.

A reduction in salt level below 1.5% or the addition of KCl\TS reduced (*p* < 0.05) cooked sausage cohesiveness (Table 5). Decreases (*p* < 0.05) in gumminess and chewiness were observed with each reduction in salt level employed, however, no significant effect on gumminess values was observed when KCl\TS was added (Table 5). This is consistent with the results reported by Gordon and Barbut [29] and Xiong et al. [55] which showed that a reduction in salt level caused a decrease in gumminess. Jimenez-Colmenero et al. [53] reported a reduction in chewiness and attributed this to a reduction in salt content. Gordon and Barbut [29] reported that replacing 1.5% NaCl with an equal ionic strength of KCl had no effect on gumminess.

### 3.7. Colour

Consumer acceptability of meat products is greatly influenced by the colour of the meat products. [56]. The colour of the cross-section of the cooked breakfast sausages is shown in Table 6. Results indicated that there were no interaction effects between factors salt level and added ingredients KCl\TS on the colour of cooked breakfast sausages.

A decrease (*p* < 0.05) in the Lightness (L-values) values of sausages was observed when KCl\TS was added; however, the salt level employed in sausages did not significantly affect lightness values (Table 6). These results differ from those reported by Fernandez et al. [50] who found an increase in lightness due to the addition of starch in pressure-treated batters. Annor-Frempong et al. [44] found that comminuted meat products containing cassava flour (from which tapioca starch is obtained) to have more acceptable internal cooked colour. They found that the fine white colour of cassava starch enhanced the desirable red colour of the meat. However, if the concentration of cassava was increased from 5.4 to 10.8%, then the colour became too pale as the redness was diluted. Sikes et al. [22] reported that the colour of HP-treated raw batters at 200 MPa was not significantly different, but pressure-treated batters were slightly whiter than untreated samples.

Samples became increasingly (*p* < 0.05) redder if salt levels were reduced from 1.5 to 1.0%, however, a further reduction to 0.5% did not significantly affect the redness of sausages (Table 6). The addition of KCl\TS decreased (*p* < 0.05) the yellowness values for cooked sausages (Table 6), however, the salt levels in sausages did not significantly affect the yellowness of sausages. Conversely, Fernandez et al. [50] found an increase in yellowness due to the addition of starch in pressure-treated batters. Wettasinghe and Shahidi [57] compared the colour of ground pork containing NaCl to pork containing a low sodium salt mixture (LSSM-57% NaCl, 28% KCl, 12% MgS04, 3% lysine.HCl) and found that there was no difference in yellowness between batches containing 1.0% NaCl or 1.0% LSSM.

In general, it is reported that colour changes in comminuted meat products can be related to water content, denaturation of the myofibrillar and sarcoplasmic proteins, changes in texture, fat content, size of fat globules, and homogeneity of the surface [21,24].

The higher the ΔE value, the simpler is to observe differences in colour; therefore, consumers can detect slight differences above ΔE = 2.0 and clear differences above ΔE = 3.5 [58]. The ΔE was not significantly affected by the salt level; however, significant (*p* < 0.05) effect on the ΔE was observed on the added ingredients (Table 6). A significant (*p* < 0.05) interactive effect between the salt level and the added ingredients was also noticed. Clear differences (ΔE ≥ 3.5) were noticed in samples containing 1% NaCl and not added ingredients (KCl\TS) and 1.5% with added ingredients (KCl\TS) indicating that consumers could have detected the colour differences.

## 4. Conclusions

The present study showed that, in general, partial replacement of NaCl with the functional ingredient KCl along with TS had a beneficial effect on the water binding abilities of the sausage batters by significantly decreasing the TEF (%) and increasing the WHC (%). Cook loss was decreased (*p* < 0.05) when KCl\TS was used with a 0.5% salt level. However, the use of KCl\TS did not have a beneficial effect on cook loss when used with 1.0% and 1.5% salt. The sensory attributes of the breakfast sausages were not significantly affected by the addition of KCl\TS. The use of KCl\TS increased sausage hardness when used with reduced salt levels (1.0%). However, independently of the salt level used, the use of KCl\TS increased (*p* < 0.05) adhesiveness and decreased (*p* < 0.05) cohesiveness. Lightness and yellowness values for cooked sausages were significantly reduced when KCl\TS was used in the formulation; however, these colour parameters were not significantly affected by salt level. Overall, results indicated that use of the functional ingredient KCl\TS has the potential to reduce sodium levels in the manufacture of low-salt breakfast sausages to 1.0%, while still possessing the organoleptic and functional properties traditionally associated with these meat products. Future research is needed to determine the microbial and physicochemical stability during storage of the developed sodium reduced sausages.

## Figures and Tables

**Table 1:** Formulation for breakfast sausages (4.0 kg) manufactured using HP-treated pork belly meat at 150 MPa for 5 min with varying salt levels and 0.25% phosphate.

Salt Level Formulation (%)	Pork Belly (kg)	Water (kg)	Seasoning (kg)	Phosphate (kg)	Salt (kg)	TS * (kg)	KCl * (kg)
0.5	2.8	0.67	0.5	0.01	0.02	-	-
0.5	2.8	0.55	0.5	0.01	0.0133	0.12	0.0067
1.0	2.8	0.65	0.5	0.01	0.04	-	-
1.0	2.8	0.53	0.5	0.01	0.0267	0.12	0.0133
1.5	2.8	0.63	0.5	0.01	0.06	-	-
1.5	2.8	0.51	0.5	0.01	0.04	0.12	0.02

* Tapiocaline and KCl were added together to batches containing each salt level. Batches were also formulated at each salt level that did not contain these ingredients.

**Table 2:** Proximate composition of raw or cooked breakfast sausages manufactured using pork belly high pressure treated at 150 MPa containing different salt levels (NaCl) and added ingredients (KCl\TS) †.

Samples	Raw Sausage Composition					Cooked Sausage Composition		
NaCl(%)/KCl\TS	Moisture (%)	Fat (%)	Protein (%)	Salt (%)	Ash (%)	Moisture (%)	Fat (%)	Protein (%)
0.5/0 *	64.40 ± 0.38 ^a^	9.81 ± 0.16 ^a^	13.42 ± 0.14 ^ab^	0.75 ± 0.02 ^a^	1.49 ± 0.01 ^a^	54.88 ± 0.91 ^a^	12.15 ± 0.28 ^a^	18.46 ± 0.10 ^a^
0.5/1	62.02 ± 0.04 ^c^	9.49 ± 0.17 ^a^	13.93 ± 0.09 ^b^	0.71 ± 0.02 ^a^	1.81 ± 0.01 ^b^	53.91 ± 0.41 ^a^	11.00 ± 0.17 ^ab^	17.39 ± 0.24 ^b^
1.0/0	63.42 ± 0.55 ^ab^	9.39 ± 0.33 ^a^	13.30 ± 0.08 ^ab^	1.35 ± 0.03 ^b^	2.06 ± 0.02 ^c^	57.13 ± 0.66 ^b^	10.44 ± 0.51 ^b^	16.51 ± 0.18 ^bc^
1.0/1	61.63 ± 0.19 ^c^	9.51 ± 0.17 ^a^	13.16 ± 0.30 ^a^	1.14 ± 0.02 ^c^	2.01 ± 0.10 ^c^	55.12 ± 0.59 ^a^	10.89 ± 0.26 ^ab^	15.94 ± 0.16 ^cd^
1.5/0	63.17 ± 0.29 ^b^	9.12 ± 0.16 ^a^	13.38 ± 0.09 ^ab^	1.67 ± 0.03 ^d^	2.64 ± 0.10 ^d^	58.48 ± 0.52 ^c^	8.62 ± 0.35 ^c^	15.69 ± 0.33 ^cd^
1.5/1	61.44 ± 0.18 ^c^	9.40 ± 0.16 ^a^	13.41 ± 0.23 ^ab^	1.69 ± 0.02 ^d^	2.61 ± 0.15 ^d^	56.57 ± 0.90 ^b^	8.30 ± 0.24 ^c^	15.25 ± 0.21 ^d^

* 0 = batches in which ingredient KCl\TS were not added and 1 = batches in which ingredient KCl\TS were added. † Data are presented as the mean ± Standard Error (*n* = 10). ^a,b,c,d^ Different letters in the same row indicate significant differences (*p* < 0.05).

**Table 3:** The effect of salt levels and added ingredients KCl\TS on the emulsion stability, water holding capacity and cook loss of breakfast sausages manufactured with pork belly meat high pressure treated at 150 MPa.

	% TEF	% Fat	WHC	Cook Loss
A: Salt Level				
0.5%	1.98 ^a^	8.69 ^a^	99.78 ^a^	21.16 ^a^
1.0%	1.83 ^a^	7.47 ^a^	99.81 ^b^	19.32 ^b^
1.5%	1.46 ^a^	7.65 ^a^	99.82 ^b^	16.13 ^c^
SL	NS	NS	0.01	0.00
B: Added Ingredients				
0 *	2.21 ^a^	7.93 ^a^	99.78 ^a^	19.50 ^a^
1	1.30 ^b^	7.94 ^a^	99.82 ^b^	18.24 ^a^
SL	0.01	NS	0.00	NS
Interactions A × B				
SL SL	NS	NS	0.04	0.01
Samples				
0.5/0	2.56	8.83	99.74	23.39
1.0/0	2.42	6.93	99.79	18.65
1.5/0	1.67	8.05	99.81	16.45
0.5/1	1.40	8.55	99.82	18.92
1.0/1	1.23	8.02	99.83	20.00
1.5/1	1.26	7.24	99.82	15.81
LSD	1.24	5.27	0.03	2.50

Data are presented as the mean (*n* = 10). TEF = Total expressible fluid. * 0 = ingredient KCl\TS were not added; 1 = Ingredient KCl\TS were added. ^a,b,c^ Different letters in the same row indicate significant differences (*p* < 0.05). SL = Significance level. NS = Not significant. LSD = least significance difference.

**Table 4:** The effect of salt levels and added ingredients KCl\TS on the sensory properties of breakfast sausages manufactured with pork belly meat high pressure treated at 150 MPa.

	Saltiness	Juiciness	Flavour	Firmness	Other Flavour	Texture	Overall Acceptability
A: Salt Level							
0.5%	1.65 ^a^	3.55 ^a^	2.37 ^a^	2.64 ^a^	2.02 ^a^	2.68 ^a^	2.20 ^a^
1.0%	2.51 ^b^	3.87 ^b^	3.11 ^b^	4.01 ^b^	1.91 ^a^	3.84 ^b^	3.41 ^b^
1.5%	3.03 ^c^	4.07 ^b^	3.55 ^c^	4.23 ^b^	1.80 ^a^	4.19 ^c^	3.83 ^c^
SL	0.00	0.00	0.00	0.00	NS	0.00	0.00
B: Added Ingredients							
0 *	2.51 ^a^	3.89 ^a^	3.12 ^a^	3.73 ^a^	1.90 ^a^	3.66 ^a^	3.26 ^a^
1	2.28 ^a^	3.77 ^a^	2.89 ^a^	3.52 ^a^	1.93 ^a^	3.50 ^a^	3.04 ^a^
SL	NS	NS	NS	NS	NS	NS	NS
Interactions A × B							
SL SL	NS	NS	NS	NS	NS	NS	NS
Samples							
0.5/0	1.62	3.50	2.31	2.69	2.00	2.70	2.36
0.5/1	1.67	3.59	2.42	2.59	2.05	2.66	2.05
1.0/0	2.67	3.92	3.34	4.03	1.92	3.89	3.47
1.0/1	2.34	3.83	2.87	3.98	1.91	3.80	3.36
1.5/0	3.25	4.25	3.70	4.84	1.78	4.37	3.97
1.5/1	2.81	3.89	3.39	3.98	1.83	4.01	3.70
LSD	0.55	0.42	0.55	0.38	0.32	0.38	0.41

Data are presented as the mean (*n* = 64). Hedonic scale: 1 = least extreme to 6 = most extreme. * 0 = ingredient KCl\TS were not added; 1 = Ingredient KCl\TS were added. ^a,b,c^ Different letters in the same row indicate significant differences (*p* < 0.05). SL = Significance level. NS = Not significant. LSD = least significance difference.

**Table 5:** The effect of salt levels and added ingredients KCl\TS on the textural properties of breakfast sausages manufactured with pork belly meat high pressure treated at 150 MPa.

	Hardness	Springiness	Adhesiveness	Cohesiveness	Gumminess	Chewiness
A: Salt Level						
0.5%	84.42 ^a^	6.90 ^a^	0.22 ^a^	0.50 ^a^	44.84 ^a^	298.22 ^a^
1.0%	103.65 ^b^	6.77 ^a^	0.26 ^b^	0.53 ^b^	54.60 ^b^	369.17 ^b^
1.5%	110.17 ^b^	6.66 ^a^	0.26 ^b^	0.58 ^c^	60.46 ^c^	400.21 ^c^
SL	0.00	NS	0.00	0.00	0.00	0.00
B: Added Ingredients						
0 *	97.42 ^a^	6.81 ^a^	0.24 ^a^	0.54 ^a^	52.48 ^a^	356.54 ^a^
1	101.41 ^a^	6.74 ^a^	0.26 ^b^	0.52 ^b^	54.10 ^a^	355.20 ^a^
SL	NS	NS	0.00	0.01	NS	NS
Interactions A × B						
SL SL	0.03	NS	NS	NS	NS	NS
Samples						
0.5/0	89.56	6.82	0.22	0.52	46.18	311.74
0.5/1	79.29	6.99	0.23	0.49	43.49	284.70
1.0/0	96.89	6.86	0.25	0.54	52.57	360.66
1.0/1	110.40	6.78	0.28	0.51	56.56	377.68
1.5/0	105.80	6.76	0.25	0.56	58.68	397.21
1.5/1	114.54	6.56	0.28	0.55	62.25	403.22
LSD	12.79	0.42	0.03	0.03	5.35	39.10

Data are presented as the mean (*n* = 10). * 0 = ingredient KCl\TS were not added; 1 = Ingredient KCl\TS were added. ^a,b,c^ Different letters in the same row indicate significant differences (*p* < 0.05). SL = Significance level. NS = Not significant. LSD = least significance difference.

**Table 6:** The effect of salt levels and added ingredients KCl\TS on the internal colour of cooked breakfast sausages manufactured with pork belly meat high pressure treated at 150 MPa.

	Lightness (L)	Redness (a)	Yellowness (b)	Total Colour Difference ΔE
A: Salt Level				
0.5%	49.51 ^a^	2.62 ^a^	12.49 ^a^	2.59
1.0%	49.19 ^a^	2.43 ^a^	12.13 ^a^	3.41
1.5%	48.87 ^a^	2.02 ^b^	11.99 ^a^	3.16
SL	NS	0.00	NS	NS
B: Added Ingredients				
0 *	50.33 ^a^	2.34 ^a^	12.48 ^a^	2.54
1	48.06 ^b^	2.37 ^a^	11.93 ^b^	3.54
SL	0.00	NS	0.01	0.02
Interactions A × B				
SL	NS	NS	NS	0.01
Samples				
0.5/0	50.02	2.64	12.59	2.36
0.5/1	49.01	2.59	12.39	2.82
1.0/0	49.60	2.49	12.31	3.52
1.0/1	48.15	2.38	11.94	3.31
1.5/0	51.36	1.89	12.52	0
1.5/1	47.03	2.14	11.46	4.49
LSD	2.21	0.28	0.76	1.18

Data are presented as the mean (*n* = 10). * 0 = Ingredients KCl\TS were not added; 1 = Ingredients KCl\TS were added. ^a,b^ Different letters in the same row indicate significant differences (*p* < 0.05). SL = Significance level. NS = Not significant. LSD = least significance difference.

## Data Availability

The datasets generated for this study are available on request to the corresponding author.
